# A New Perspective on the Renin-Angiotensin System

**DOI:** 10.3390/diagnostics13010016

**Published:** 2022-12-21

**Authors:** Adrian Martyniak, Przemysław J. Tomasik

**Affiliations:** Department of Clinical Biochemistry, Institute of Pediatrics, Jagiellonian University Medical College, 30-663 Krakow, Poland

**Keywords:** hypertension, cardiovascular disease, renin, aldosterone, angiotensin A, angiotensin 1–7, alamandine, angiotensin convertase type II

## Abstract

Cardiovascular disease (CVD) is the leading cause of death in the world. Hypertension is a serious medical problem not only in adults but also in children and adolescents. The renin-angiotensin-aldosterone system (RAAS) is one of the most important mechanisms regulating blood pressure and the balance of water and electrolytes. According to the latest reports, RAAS acts not only on endocrine but also on paracrine, autocrine, and intracrine. Moreover, RAAS has a component associated with hypotension and cardioprotective effects. These components are called alternative pathways of RAAS. The most important peptide of the alternative pathway is Ang 1–7, which is related to the Mas receptor. Mas receptors have widely known antihypertension properties, including vasodilatation, the release of nitric oxide, and increased production of anti-inflammatory cytokines. Another interesting peptide is angiotensin A, which combines the properties of the classical and alternative pathways. No less important components of RAAS are the proteolytic enzymes angiotensin convertase enzyme type 1 and 2. They are responsible for the functioning of the RAAS system and are a hypertension therapeutic target. Also involved are tissue-specific enzymes that form a local renin-angiotensin system. Currently, a combination of drugs is used in hypertension treatment. These drugs have many undesirable side effects that cannot always be avoided. For this reason, new treatments are being sought, and the greatest hope comes from the ACE2/ang 1–7/MasR axis.

## 1. Introduction

According to the WHO in 2020, cardiovascular disease (CVD) is the leading cause of death in the world. The WHO report indicates that the two most common causes of death are ischemic heart disease and stroke. Together, they account for 27% of the deaths, respectively, 16% and 11%. The CDC warns that 1 in every 5 deaths in the US is caused by CVD [1]. One of the main causes of CVD is arterial hypertension (AH), which is directly or indirectly responsible for 47% of myocardial ischemia and 54% of strokes. According to estimates, 1 in 3 adults in the US has hypertension [2]. Hypertension is a serious medical problem not only in adults but also in children and adolescents. It is estimated that, in the United States, depending on the criteria, AH is associated with 0.3 to 4.5% of the pediatric population [2]. The etiology of primary AH is unknown. Genetic and environmental factors are assumed to have the greatest influence. Furthermore, increased cardiac output increases peripheral resistance, or a combination of both causes an increase in arterial blood pressure. With the use of modern diagnostic methods, it is often impossible to find the direct cause of high arterial pressure. Many processes and systems are responsible for the efficient functioning of the cardiovascular system, which can be divided into hemodynamic, nervous, humoral, and renal. One of the most important humoral systems that regulate arterial blood pressure is the renin-angiotensin-aldosterone system (RAAS) [3].

## 2. The Role of RAAS in Regulating Arterial Blood Pressure

**Figure 1:**
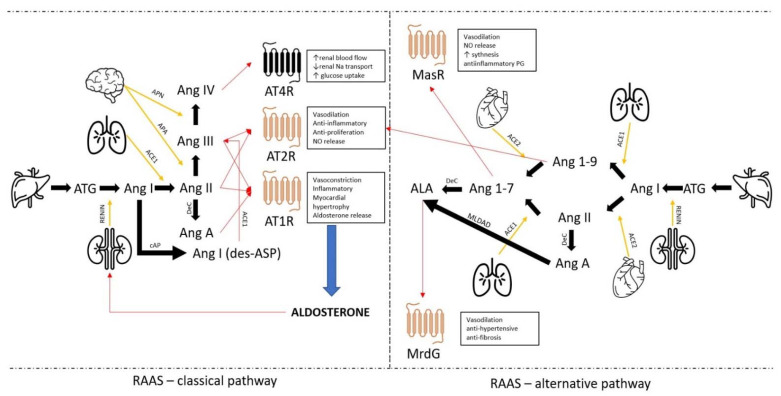
Scheme of the classical and alternative pathways of RAAS. Orange receptors represent a G-protein coupled receptor family, black receptors represent an insulin-regulated membrane aminopeptidase. Yellow arrows represented a tissue-specific enzyme, and red arrows represented the direction of peptide actions. ATG—angiotensinogen; Ang I—angiotensin I; Ang II—angiotensin II; Ang III—angiotensin III; Ang IV—angiotensin IV; Ang A—angiotensin A; AT1R—angiotensin receptor type 1; AT2R—angiotensin receptor type 2; AT4R—angiotensin receptor type 4; APN—aminopeptidase N; APA—aminopeptidase; cAP—circulation aminopeptidase; Dec—decarboxylation; ACE1—angiotensin convertase enzyme type 1; ACE2—angiotensin convertase enzyme type 2; Ang 1–9—angiotensin 1–9; Ang 1–7 angiotensin 1–7; ALA—alamandine; MasR—Mas receptor; MrdG-Mas-related G-coupled receptor type D; MLDAD—mononuclear leukocyte-derived aspartate decarboxylase; PG—prostaglandin; NO—nitro oxide.

RAAS is one of the most important mechanisms regulating blood pressure and the balance of water and electrolytes in the body by changing the sodium concentration in plasma and the volume of extracellular fluid [4]. RAAS consists of interrelated elements, including a hormone with enzymatic activity-renin, angiotensin peptides with various activities, and a mineral corticosteroid-aldosterone [5]. The RAAS can act systemically and locally. The systemic action is to regulate arterial blood pressure and sodium concentration by the kidneys [6]. Currently, more emphasis is placed on understanding local autonomous RAAS. According to the latest reports, RAAS acts not only on endocrine but also on paracrine, autocrine, and intracrine [7]. It has also been shown that RAAS has a component with an opposite effect, which means it is able to lower the pressure through short fragments of angiotensin with an antagonistic effect against the peptides of the classical pathway. These components are called alternative pathways of RAAS. Angiotensin 1–7 (Ang 1–7), angiotensin 1–9 (Ang 1–9), and alamandine (ALA) are recognized as alternative pathway peptides with antihypertensive effects [8,9,10]. The advancement of knowledge about RAAS is also changing our understanding of hypertension. Primary arterial hypertension may result not only from RAAS overactivity in increasing arterial blood pressure but also from inhibition of contrary mechanisms associated with classical RAAS and now recognized as an integral part of it. Figure 1 shows a schematic division of RAAS into classical and alternative pathways, as well as the peptides and enzymes involved in their regulation. There are also autonomous renin-angiotensin systems that do not act on aldosterone, so the term “renin-angiotensin system” (RAS) is used. RAS operates within a tissue/organ, locally controlling blood flow or regulating inflammatory processes by inhibiting or activating cytokine releases. Tissues and organs capable of autonomous regulation include the brain, heart, kidneys, pancreas, and adipose tissue. The ability to de novo synthesize all components of the RAS is the main condition for the existence of a local system. The local system includes tissue-specific enzymes such as chymases in the heart and peptides of angiotensin derivatives. In recent years, many new peptides and enzymes related to the RAAS have been discovered.

### 2.1. Renin

The functioning of the RAAS depends on the activity of renin in the body. Impaired renal renin synthesis and secretion are the main causes of the expansion of blood volume, high arterial blood pressure, and organ damage [11]. Renin is synthesized and secreted by cells of the juxtaglomerular apparatus, located in the afferent artery of the kidneys. The main factor determining the amount of renin produced is the transcription rate of the renin gene. Renin is an enzyme that belongs to the proteases. It is also known as angiotensinase and consists of 340 amino acid residues. Renin is produced by the action of intracellular proteolytic enzymes on the inactive form, prorenin [12]. Under physiological conditions, up to 80% of the prorenin produced is secreted outside the cell in an unchanged form. Prorenin has very low enzymatic activity and, to the best of our knowledge, is not converted to renin extracellularly [11]. Renin secretion is controlled by several systems, and most of the secretion occurs under stimulation of the β-adrenergic system [13]. Renin secretion is also under the control of baroreceptors, which can be divided into arterial baroreceptors that control renin secretion by α-adrenergic receptors located in the blood vessel wall, responding to changes in blood flow and pressure on the vessel wall and intrarenal baroreceptors [11]. Intrarenal baroreceptors respond to decreased intrarenal blood flow by increasing renin secretion. Renin secretion is also influenced by prostaglandins synthesized by the kidneys in large amounts. Renin release also depends on feedback, and angiotensin II is a strong inhibitor. Renin secretion is under the control of the cytoplasmic calcium concentration in cells of the juxtaglomerular apparatus. The increase in calcium concentration in the cytosol of cells of the juxtaglomerular apparatus inhibits renin release [14]. Renin catalyzes the transformation of angiotensinogen into angiotensin I.

### 2.2. Angiotensinogen and Related Peptides

The only precursor to all angiotensin peptides is angiotensinogen (AGT). AGT consists of 485 amino acid residues, of which 33 AA form the signal sequence. ATG synthesis occurs mainly in hepatocytes. In a smaller amount, ATG is synthesized and released locally by the heart, kidneys, brain, or adrenal glands, as well as adipocytes and the endothelium of blood vessels. Adipocytes are the second, after hepatocytes, source of AGT [15]. AGT is a member of the non-inhibitory serpin (serine protease inhibitor) superfamily. The same group of proteins includes alpha 1 antitrypsin, alpha 1 antichymotrypsin, and antithrombin III. ATG production is mainly controlled by hormones [16]. Estrogens, steroids, and thyroid hormones stimulate ATG production [17]. The amount of ATG formed is determined by the rate of gene transcription. Angiotensin II has been shown to increase the stability of AGT mRNA. This has positive feedback on AGT. The opposite situation occurs in the case of renin, where an increase in ATG concentration inhibits the activity of renin [18]. Directly after synthesis, the AGT protein is secreted extracellularly. After enzymatic cleavage of the ten amino acid peptides of Ang I from AGT, the remaining molecule is called des (Ang I) AGT. Although des (Ang I) AGT contains 98% of the parent protein sequence, its function within the body and its biological properties are unknown [17]. However, it is now known that, in addition to renin, many other enzymes such as cathepsin D, cathepsin G, kallikrein, pepsin, tissue plasminogen activator, tonin, and trypsin convert AGT to Ang I or Ang II. These enzymes are abundant in tissues or some types of cells, which has led to speculation that these enzymes may have a local modulating effect on the activity of the RAAS [19].

Ang I decapeptide (Asp-Arg-Val-Tyr-Ile-His-Pro-Phe-His-Leu) is produced by renin cleavage of the N-terminal fragment of AGT. It has no significant biological activity. It serves primarily as a substrate for the production of other biologically active peptides such as angiotensin II, angiotensin III, angiotensin IV, and angiotensin 1–7 or angiotensin 1–9 [20].

### 2.3. Aldosterone

Aldosterone is a hormone of the adrenal cortex that regulates the balance of water and electrolytes. Recent studies indicate that primary aldosteronism is a common cause of hypertension, with an incidence of 5–10% in patients with general hypertension and approximately 20% in severe and refractory hypertension [21,22]. The primary action of aldosterone is to induce sodium and water retention and increase blood volume. This effect occurs secondary to the stimulation of cytoplasmic mineralocorticoid receptors in distal renal tubular cells, increasing the activity and number of epithelial sodium channels, which promotes unidirectional transepithelial sodium transport [23,24]. Human studies suggest that excess aldosterone has a direct pro-inflammatory and fibrotic effect, contributing to target organ degradation, which manifests itself in the development of vasculitis, kidney and heart inflammation, fibrosis, and hypertrophy [25].

These three elements are common to the RAAS; further development towards the classical or alternative pathways is dependent on enzymatic activity.

## 3. RAAS Classical Pathway

### 3.1. Angiotensin Convertase Enzyme Type 1 (ACE1)

ACE1 is classified as an enzyme of the group of dipeptidases and endoproteases. Its main effect is the hydrolysis of angiotensin I into angiotensin II by cleaving 2 AA from the C-terminus of the peptide. The ACE1 glycoprotein is associated with the cell membrane of the vascular endothelial cells of the lungs, where it is most active, and of the proximal renal tubules and neuroepithelial cells. The activity of the enzyme depends on the presence of chloride ions, while it is inhibited by chelating agents such as heavy metals, sulfhydryl compounds, and some peptides [26]. An additional function of the enzyme is the inactivation of vasodilating peptides: bradykinin and kallidin [27], which further enhances the hypertensive effect. ACE1 also hydrolyzes a wide range of other vasoactive peptides, such as substance P, enkephalin, and neurotensin [28].

### 3.2. Angiotensin II

Angiotensin II (Asp-Arg-Val-Tyr-Ile-His-Pro-Phe) has the strongest biological activity among the known hypertensive angiotensin-related peptides. The action of this octapeptide is based on its interaction with type I and type II angiotensin receptors. The peptide has a stronger affinity for the angiotensin type I receptor (AT1R), which is located mainly in the kidneys, vascular smooth muscle, lungs, and liver, and a lower affinity for the angiotensin type II receptor (AT2R), which is expressed primarily in the prenatal period and later in childhood. In adults, the receptor is present only in the kidneys, heart, and blood vessels [29]. Both receptors belong to the superfamily of G protein-bound receptors with antagonistic properties. The effects of AT1R stimulation may be vasoconstriction, activation of inflammatory processes, myocardial hypertrophy and fibrosis, and the release of aldosterone from the adrenal cortex, while activation of AT2R results in vasodilation of blood vessels, inhibition of cell proliferation, myocyte hypertrophy, and nitric oxide release.

### 3.3. Angiotensin III

Angiotensin III (Ang III) (Arg-Val-Tyr-Ile-His-Pro-Phe) is a peptide deriving directly from angiotensin II with the participation of aminopeptidase A (APA) [30]. There is also a second pathway for peptide synthesis. Angiotensin III can also be formed directly from Ang I. First, circulating aminopeptidases break down Ang I to Ang I (des-ASP). The peptide is then degraded into Ang III by the ACEI [31]. APA cleaves the N-terminal amino acid to form a heptapeptide with properties very similar to those of Ang II. Like Ang II, the peptide affects the ATR1 and ATR2 receptors. Some researchers have promoted the hypothesis that Ang III is the main effector peptide of the RAAS. This is supported by the rate of conversion of Ang II to Ang III, the relatively short half-life of Ang II, and the interaction with the AT1R and AT2R. However, this hypothesis requires further research, mainly on the human model [32,33,34]. In another study on an animal model, intracerebral injection of Ang II or Ang III showed a similar increase in arterial blood pressure, and the cerebral form of APA could be a pharmacological target for the treatment of hypertension [35,36]. However, peripherally, the power of Ang III is equivalent to that of Ang II for increasing arterial blood pressure, aldosterone secretion, and renal function [37,38]. However, it may differ in terms of its effect on cell proliferation or inflammation regulation [38].

### 3.4. Angiotensin IV

Angiotensin IV (Ang IV), according to contemporary studies, has rather low hypotension activity but shows high activity in other systems. It is formed from Ang III by the aminopeptidase N (APN) action. Ang IV acts through a specific ATR4 receptor. The ATR4 receptor is widely distributed and is found in many tissues, including the brain, adrenal glands, kidneys, lungs, and heart. In the kidneys, Ang IV increases blood flow in the renal cortex and reduces the transport of sodium ions into the isolated proximal tubules of the kidney. The ATR4 receptor has been identified as a transmembrane enzyme, insulin-regulated membrane aminopeptidase (IRAP). IRAP is an integral type II membrane protein belonging to the M1 aminopeptidase family and is found mainly in GLUT4 vesicles in insulin-sensitive cells [39]. ATR4 improves cognitive functions and cell signal transmission and is anti-inflammatory [40]. The achievable effect is possible, among others, due to increased glucose access to cells, which will provide ATR4. However, the effect of arterial blood pressure on Ang IV is minimal.

### 3.5. Other Important Enzymes-Aminopeptidase A and N

Both enzymes are membrane-bound zinc metallopeptidases. APA hydrolyzes the cleavage of the N-terminal aspartate from Ang II to form Ang III, and then APA hydrolyzes the cleavage of the N-terminal arginine from Ang III to form Ang IV. Both peptides are active in the brain, where they form the local RAS. The increase in arterial blood pressure occurs because of the activation of several mechanisms, including increased salt intake, thirst, and the release of pituitary vasopressin [41,42,43]. Most of these effects are caused by the AT1R receptor. Studies in rats have shown that locally produced Ang III in the brain has a stronger hypertensive effect than peripheral Ang II [44]. It is suggested that specific and selective inhibitors of APA and APN may be a potential therapeutic target in the treatment of hypertension [45].

### 3.6. Angiotensin A

Angiotensin A is a unique peptide. On the one hand, it presents hypertensive properties in the classical pathway. On the other hand, it indirectly presents the antihypertensive properties in the alternative pathway. Structurally, it resembles angiotensin II, as they differ by only one amino acid. However, the substitution of alanine for N-terminal asparagine in this octapeptide creates a peptide with altered properties [46]. Ang A is formed from angiotensin II by decarboxylation of Asp1. Decarboxylation is performed in human mononuclear lymphocytes. Ang A is physiologically present in the circulation at a concentration less than 20% of the Ang II concentration [47]. Ang A interacts with the AT1R and AT2R. Ang A, compared to Ang II, has a similar affinity to AT1R. The affinity for the AT2R is not well defined. Jankowski et al. suggested in their work that Ang A has an increased affinity for the AT2R as compared to Ang II [46]. Such a situation would qualify this peptide for the group of antihypertensive peptides. The study by Yang et al. suggests that both peptides have a similar affinity to AT2R [47]. In animals, Ang A administration resulted in limited vasoconstriction compared to Ang II and an increased proliferative effect on vascular smooth muscle cells than Ang II [48,49]. The observed concentrations of the peptide, along with its limited activity, suggest a slight influence of the peptide on pressure regulation. However, this peptide is somewhat suspended between the hypertensive and hypotensive effects because it serves as a substrate for the synthesis of alamandine.

## 4. Alternative Pathway of RAAS

### 4.1. Angiotensin 1–7

Angiotensin 1–7 (Asp-Arg-Val-Tyr-Ile-His-Pro) is produced mainly by the action of the enzyme angiotensin-converting enzyme type II (ACE2) directly from Ang II. Other enzymes, such as angiotensin-converting enzyme type I (from angiotensin 1–9), proline endopeptidase, neprilysin, or proline carboxypeptidase (from other angiotensin derivatives), also participate in the formation of Ang 1–7. Ang 1–7 is the selective endogenous ligand for specific Mas receptors (MasR) [49]. This receptor, along with angiotensin receptors (AT1R and AT2R), belongs to the G protein-coupled receptor family. The location of the Mas receptor on the endothelium of blood vessels, macrophages, and neurons suggests involvement in the local renin-angiotensin systems. The Ang 1–7 lowers arterial blood pressure (and is antagonistic to classic RAAS) due to vasodilation of blood vessels, induction of the synthesis of anti-inflammatory prostaglandins, and increases in the release of nitric oxide.

Ang 1–7 also has a positive effect on heart muscle by limiting the proliferation and hypertrophy of cardiomyocytes. The peptide significantly affects the regeneration and remodeling of coronary vessels. The effects on the kidneys include the regulation of sodium transport and an increase in glucose resorption. Ang 1–7 can regulate lipid metabolism and protect against insulin resistance [50].

### 4.2. Angiotensin 1–9

Angiotensin 1–9 (Asp-Arg-Val-Tyr-Ile-His-Pro-Phe-His) is formed from angiotensin I in a reaction catalyzed by ACE2 or by tissue-specific enzymes such as cathepsin A in cardiac tissue. The presence of Ang 1–9 has been found in many tissues and organs, including the heart, kidneys, or testes. The highest concentrations are observed in the endothelium of the coronary vessels. Angiotensin 1–9 has a very similar effect to angiotensin 1–7; however, Ang 1–9 has a stronger effect on heart tissue than on the blood vessel system [51]. Angiotensin 1–9 acts through AT2R. Ang 1–7 can be formed directly from Ang 1–9 by angiotensin-converting enzyme type 1 and neprilysin.

### 4.3. Alamandine

Alamandine (ALA) and angiotensin 1–7 have nearly identical amino acid sequences. They differ only in the amino acid Asp/Ala at the N-terminus of the chain (Ala-Arg-Val-Tyr-Ile-His-Pro). Alamandine is formed by the catalytic hydrolysis of Ang A by ACE2 or by the decarboxylation of ASP in the Ang 1–7 molecule. The similar structure of both peptides explains their similar biological properties. In animal models (mice), ALA caused endothelial-dependent relaxation of blood vessels in the aortic regions. Microinjections of ALA into the region of the brain responsible for hypotensive cardiovascular effects similar to those exhibited by Ang 1–7 [52]. ALA can even counteract the vasoconstriction induced by its precursor, Ang A, without affecting the vasoconstriction induced by Ang II [21]. In relation to Ang 1–7, alamandine is devoid of antiproliferative activity. Despite the similar structure of both peptides, it has been proven that alamandine acts through receptors other than Ang 1–7 act. Up until today, it is known that ALA is a ligand for Mas-related G-coupled receptor type D (MrgD), whose expression has been observed in the nervous system, specifically in nociceptive neurons, muscles, the heart, and testes [52,53,54].

### 4.4. Angiotensin Convertase Enzyme Type II (ACE2)

ACE2 is an enzyme from the group of monocarboxypeptidases expressed on the surface of cell membranes. The catalytic domain of the enzyme is exposed to the circulation, where it catalyzes the hydrolysis of angiotensin peptides. It is found primarily in the cardiovascular system, intestines, lungs, and kidneys. In the cardiovascular system, ACE2 is expressed in cardiomyocytes, epicardial adipose tissue, cardiac fibroblasts, smooth sarcoma, and the vascular endothelium [55,56]. Currently, the two main activities of the enzyme are defined. First, it is an endogenous RAAS regulatory enzyme; second, the enzyme is a cellular receptor for the SARS-CoV and SARS-CoV-2 viruses [57].

The discovery of ACE2 changed the understanding of blood pressure regulation. It is currently believed that the ACE2/ang17/MasR axis is the second arm of the balance and protects against the negative effects of overactivity of the ACE1/Ang II/AT1R axis. ACE2 protects against RAS-induced injury through two processes: (1) degradation of angiotensin I and angiotensin II to limit substrate availability on the unfavorable ACE/angiotensin II/AT1R axis and (2) production of angiotensin 1–7 to increase substrate availability on the protective axis of the ACE2/angiotensin 1–7/MasR [58].

## 5. Local Renin-Angiotensin System-Role of Angiotensin’s Peptides in Heart Failure

Angiotensin peptides and related enzymes play a critical role in the pathogenesis of heart failure [HF]. A very important role is played in the activity of ACE1 and ACE2. In this concept, the balance is maintained by the activity of the ACE1/AngII and ACE2/Ang1–7 axes. Although the role of the AEC1/AngII axis is widely known and accepted, the role of the ACE2/Ang1–7 axis is under constant discussion. Especially in patients who are already treated for AH or HF.

In the treatment of HT, drugs are used as in the treatment of AH. ACEI remains the first-line therapy for all patients with a reduced ejection fraction. Sartans may be used instead of ACEI in patients with intolerance or in conjunction with ACEI to further reduce symptoms. Currently, a combination of both drugs is often used [59]. Although these HF pharmacotherapies are beneficial, patients with HF continue to be plagued by clinical deterioration, high morbidity, and mortality [60]. Regardless of the ability of ACEI to inhibit the effects of ACE, Ang II levels may remain elevated in optimally treated patients with HF. Approximately 50% of patients on ongoing ACEI therapy have elevated levels of Ang II as a result of activation of mast cell chymase, so a problem for patients treated with ACEI may be local synthesis of Ang II. Therefore, other ways to reduce the harmful effects of Ang II are sought. The natural ACE inhibitor is ACE2. Efforts to inhibit ACE activity have been found to be insufficient. Therefore, the focus has been on pharmacologically enhancing ACE2 activity [55]. A well-researched tool to enhance the effects of ACE2 is recombinant human ACE2 (rhACE2). A randomized, double-blind, placebo-controlled study administered rhACE2 intravenously to healthy subjects and found that rhACE2 was well tolerated. Despite significant changes in angiotensin peptide concentration, hypotension did not occur, suggesting the presence of effective compensatory mechanisms in healthy volunteers [61].

Biomarkers are used to measure the extent of heart damage. One of the most frequently chosen markers of heart failure is N-terminal prohormone brain natriuretic peptide (NT-proBNP). NT-proBNP is a prohormone secreted mainly by left ventricular cardiomyocytes that participates in maintaining cardiovascular homeostasis. The main functions of the active peptide are increased glomerular filtration, decreased sodium reabsorption in the kidney, inhibition of aldosterone secretion, and decreased sympathetic system activity [62]. The potential action of NT-proBNP coincides with the action of the alternative RAA pathway. Short-term neurohormonal activation has a positive effect on the cardiovascular system; however, with chronic activation, these responses result in hemodynamic stress and exert deleterious effects on the heart and the circulation. The increased sympathetic activity and the action of NT-proBNP lead to the release of renin by the glomerular apparatus and thus to the activation of the RAA. Research confirms that short classical pathway peptides such as Ang II, Ang III, or Ang IV have a negative effect on the heart. The alternative route peptide Ang 1–7 is expected to counteract the effects of angiotensin II and weaken left ventricular (LV) remodeling [63,64].

## 6. Novels and New Perspective on RAAS

Currently, a combination of drugs such as angiotensin-converting enzyme inhibitors (ACEI), angiotensin receptor blockers (sartans), and calcium channel blockers are used in hypertension treatment. These drugs have many undesirable side effects that cannot always be avoided. Setting the right dose of drugs and their possible combinations is a laborious and time-consuming process. In addition, it exposes the patient to additional stress and increases the cost of treatment. The laboratory test currently used that helps to initially differentiate the source of hypertension and thus supports treatment is the determination of plasma renin activity (PRA). Unfortunately, the test itself is imprecise, and the result can be interpreted in several ways. Increased PRA values are observed, for example, in some cases of primary hypertension, renal artery stenosis, and reninoma. Decreased values, in turn, may suggest primary hyperaldosteronism or a defect in renin synthesis or secretion. The medications taken by the patient are also important. Drugs for hypertension, β-blockers, and α-agonists have been proven to reduce PRA values. Diuretics, oral contraceptives, or, for example, ACEIs, can in turn cause elevated results. The preanalytic phase, patient preparation, sample collection, and transport to the laboratory are very important. Failure to meet any of the conditions will result in false results.

New methods of treatment and diagnostics for arterial hypertension are being sought. The greatest hopes are associated with a new group of drugs acting on the ACE2/1–7/MasR axis [65]. These drugs, which are essentially agonists of the Mas receptor, would enhance the positive effect of angiotensins 1–7 on the human body. The problem is the inability to verify the effects of these drugs at the capture point. The currently used immunologically-based diagnostic methods for arterial hypertension cannot provide adequate sensitivity for the determination of short fragments of angiotensin such as angiotensin 1–7 or Ang 1–9. The crosslinking and specificity interference of the antibodies are also big obstacles. However, mass spectrometry gives diagnostic possibilities for the fast and very reliable determination of short angiotensin fragments [66].

In 2013, PARADIGM-HF researchers conducted a randomized, double-blind, parallel group, active-controlled, two-arm, event-driven trial comparing the long-term efficacy and safety of enalapril (ACEI) and LCZ696, one of the new group drugs, angiotensin receptor neprilysin inhibitors (ARNI). This class of drugs was developed to block RAAS and augment natriuretic peptides. ARNIs have the potential to favorably modulate the neurohormonal imbalance that characterizes heart failure. In this group, the blockade of RAA is achieved by the AT1R antagonism, and not by ACEI. It is hypothesized that the risk of angioedema will not increase. The results of the study turned out to be in line with expectations, and LCZ696 will be determined as an alternative to ACEI in patients with HF [67]. In 2018, another study was conducted. The 5-year estimated number needed to treat patients with ARNI therapy incremental to ACEI therapy overall and for clinically relevant subpopulations of patients with HF are comparable with those for well-established HF and AH therapeutics. According to the authors of the cited study, these data further support guideline recommendations for the use of ARNI therapy among eligible patients with HFrEF [68].

## 7. Conclusions

The RAAS has been known for more than 100 years. Emerging reports of new peptides, receptors, and regulatory pathways lead us to believe that this system is more complete than the original research suggested. RAAS is a mixture of enzymes and peptides that control arterial blood pressure. The multitude of elements causes RAAS to be prone to dysregulation. The increased incidence of arterial hypertension due to avalanches and the growing public awareness of its negative effects can cause pressure to better understand the regulatory mechanisms. Now, researchers are looking for new drugs and strategies to treat hypertension faster and more effectively, while at the same time reducing the side effects of drugs. The key to understanding the RAAS and finding the right therapy may be knowing the exact concentrations of the panel of angiotensin peptides in the human body. Treatment in the form of RAS blockers has not been very effective so far in some patients, especially in African Americans (33% of this population has hypertension), in whom high arterial blood pressure is accompanied by low levels of renin (decrease in systemic RAS activity), high levels of vasopressin, and susceptibility to salt in the diet. Monotherapy for the treatment of hypertension is ineffective in more than half of all cases, and responses to a compound, regardless of its chemical family, vary widely from person to person. Therefore, monitoring therapy, such as measuring the concentration of active angiotensin fragments, can bring measurable benefits to patients.

Identifying Ang A/almandine-MrgD and Ang 1–7/MasR signaling cascades is the latest step in understanding the complexity of RAS, their role in cardiovascular physiology and pathology, and potential establishing points of modulation for treatment. This signaling pathway connects to both the “harmful” and “protective” axes of RAS, and Ang A is located at the “junction” of the system because it can either directly induce vasoconstrictor or pro-proliferative effects or indirectly induce opposing effects after further metabolism of alamandine. A very promising new method of the treatment of arterial hypertension seems to be alamandine, which can be considered the central molecule of the signaling cascade. Alamandine appears to antagonize the effects induced by Ang A, leading to a negative feedback loop. Alamandine can also be generated from both “harmful” Ang A and “protective” Ang 1–7.

Laboratory diagnosis of arterial hypertension is currently based on two pillars: basic biochemical parameters from urine and serum, such as electrolytes or creatinine, allowing the evaluation of kidney function and hormone studies—PRA and aldosterone. Thanks to these tests, we are able to determine whether arterial hypertension originates in the reninium component or the sodium-volume component. Depending on the results, the appropriate treatment regimen for the patients is established. Doing so made sense a few years ago, when knowledge about the RAAS was quite limited. Currently, the search is focused on specific capture points such as ACE2, MrgD, MasR, or AT2R. The new drugs will be able to precisely control individual pathways of the RAA system, and the number of side effects will be significantly reduced. To achieve the intended effect, it is also necessary to extend the diagnostics. As mentioned above, high hopes are placed on determining angiotensin fragments by mass spectrometry. Despite the first successes and the development of commercial kits, the preanalytical phase and proper patient preparation remain a problem.

## Data Availability

Not applicable.
